# Screening of *CYP1B1* and *LTBP2* genes in Saudi families with primary congenital glaucoma: Genotype-phenotype correlation

**Published:** 2011-11-12

**Authors:** Khaled K. Abu-Amero, Essam A. Osman, Ahmed Mousa, Joshua Wheeler, Benjamin Whigham, R. Rand Allingham, Michael A. Hauser, Saleh A. Al-Obeidan

**Affiliations:** 1 Ophthalmic Genetics Laboratory, Department of Ophthalmology, College of Medicine, King Saud University, Riyadh, Saudi Arabia; 2 Glaucoma Unit, College of Medicine, King Saud University, Riyadh, Saudi Arabia; 3 Department of Ophthalmology, College of Medicine, King Saud University, Riyadh, Saudi Arabia; 4 Center for Human Genetics, Duke University Medical Center, Durham, NC; 5 Department of Ophthalmology, Duke University Eye Center, Durham, NC; 6 Department of Ophthalmology, College of Medicine, University of Florida, Jacksonville, FL

## Abstract

**Purpose:**

Primary congenital glaucoma (PCG) is a severe form of glaucoma that presents early in life. PCG is a clinical and genetic entity that is distinct from juvenile forms of glaucoma. Inheritance is usually autosomal recessive and therefore the disease might be more common in societies where consanguinity is high. We studied the prevalence of cytochrome P450, family 1, subfamily B, polypeptide 1 (*CYP1B1*) and latent-transforming growth factor beta-binding protein 2 (*LTBP2*) mutations in a group of Saudi PCG patients and attempted to correlate the mutation status with the disease severity.

**Methods:**

Genomic DNA was collected from 54 unrelated Saudi PCG families (74 patients) who were diagnosed as having PCG by standard ophthalmological examinations and screened for mutations in *CYP1B1* and *LTBP2* by sequencing. We also examined the effect of mutations on the phenotype of patients with PCG (phenotype-genotype correlation).

**Results:**

Mutations in *CYP1B1* were identified in 41 (75.9%) of affected patients. No mutation in *CYP1B1* was found in 13 (24.1%) affected persons. We detected a total of 13 mutations: 9 missense mutations (G61E, A119S, R390H, P437L, D441G, A443G, G466S, G466D, and R469W), 2 deletions (g.4238_4247del and g.7901_7913del), and 2 nonsense mutations (R355X and R444X). Two mutations, G466S and D441G, were novel. The G61E mutation was by far the most common mutation detected. PCG cases with *CYP1B1* mutation(s) presented with a high degree of haze and greater cup/disc ratio than those with no mutation(s). Also, PCG cases with a mutation had higher post operative indices in terms of post operative haze and the need for anti-glaucoma medications. Additionally, the surgical success rate was higher 13/14 (92.9%) among cases without mutation than those with mutation 42/60 (70%). No mutation(s) were found in *LTBP2* in any of the tested patients.

**Conclusions:**

*CYP1B1* mutations are the predominant cause of PCG in the Saudi Arabian population with G61E as the dominant disease-associated allele. PCG cases with a mutation had higher last postoperative visit indices in terms of postoperative haze and the need for anti-glaucoma medications. This will be a valuable parameter in predicting disease severity earlier on and might help in predicting the surgical outcome.

## Introduction

Primary congenital glaucoma (PCG; OMIM 231300) is a severe form of glaucoma that presents early in life [1]. PCG is a clinical and genetic entity that is distinct from juvenile forms of glaucoma [2,3]. Despite its rarity, PCG and other forms of childhood glaucoma were once the leading cause of admission to schools for the blind in the United States in the first part of the 20th Century [4]. PCG results from developmental abnormalities (trabeculodysgenesis) that affect the aqueous humor outflow pathway. These changes cause elevated intraocular pressure (IOP) and secondary glaucomatous optic nerve damage [5]. PCG clinical features include elevated IOP, corneal edema, enlargement of the globe (buphthalmos), corneal enlargement, rupture of Descemet’s membrane (Haab’s Striae), and optic nerve damage [6]. Additional clinical features include, photophobia, ephiphora, and blepharospasm [7].

The prevalence of PCG ranges widely. The highest prevalence is found in the Gypsy population of Slovakia where 1 in 1,250 is affected. The prevalence of PCG is 1 in 2,500 among Saudi Arabians [8]. In Western populations the prevalence is approximately 1 in 20,000 [9,10]. The high rate of consanguinity among Slovakian Gypsies and Saudi Arabians accounts for the increased prevalence of PCG in these populations. In Southern India, the prevalence of PCG is estimated at 1 in 3,300 live births accounting for 4.2% of overall childhood blindness [11]. In a retrospective study covering the period from 2006 to 2010, the Glaucoma unit at King Abdulaziz University Hospital (KAUH, Riyadh, Saudi Arabia) where approximately 600 new glaucoma patients are seen annually, pediatric glaucoma accounts for about 3% of which 2.7% are PCG.

Two genes have been reported to cause PCG, *CYP1B1* (OMIM *601771; cytochrome P450, subfamily I, polypeptide I) and *LTBP2* (OMIM *602091; latent transforming growth factor beta binding protein 2) [7,12]. Both genes cause a recessive form of this disease. *CYP1B1* is the most common identifiable cause of PCG. There are over 70 reported disease-associated mutations in *CYP1B1*. In Saudi Arabia, *CYP1B1* mutations account for 96% of PCG cases [6,8]. *LTBP2* is a much rarer cause of PCG, being reported only in Pakistan and European gypsies [13].

This study was conducted to examine the role of *CYP1B1 and LTBP2* in the Saudi Arabian population and to examine the effect of mutations on the phenotype of patients with PCG and the influence of the mutation on the surgical outcome.

## Methods

### Patients

This research adhered to the tenets of the Declaration of Helsinki. All participants or their legal guardians signed an informed consent after being informed about the nature of the research. The study was approved by the ethical committee (proposal number # 08–657) of College of Medicine at King Saud University, Riyadh, Saudi Arabia. All study subjects were self identified as Saudi Arabian ethnicity. Family names were all present in the database of Arab families of Saudi Arabian origin. Additionally, these names indicated that all five major Saudi Arabian provinces were represented in the study population. Expatriates were excluded from this study. Subjects with clinically diagnosed PCG and healthy controls were recruited into the study at the glaucoma clinic of King Abdulaziz University Hospital (KAUH) in Riyadh, Saudi Arabia. All patients were unrelated to each other and never participated in a similar study or genetically tested. Controls were unrelated to the patients and to each other. All patients underwent complete eye examination under general anesthesia. Examination included assessment of intraocular pressure (IOP) with Perkins tonometer and/or Tonopen, anterior segment examination using a portable slit lamp, measurement of corneal diameter, and gonioscopy. Dilated fundus examination for optic disc evaluation was performed with an indirect and direct ophthalmoscope. Inclusion criteria were: increased corneal diameter (>12.0 mm), raised IOP (>21 mHg under sedation) and optic disc changes when clarity of the media permitted. Combined trabeculectomy and trabeculotomy versus nonpenetrating deep sclerectomy with adjuvant mitomycin C were the initial surgical procedures. Patients associated with other ocular or systemic anomalies were excluded. Non glaucomatous controls (n=50) were recruited from KAUH. Entry criteria for those subjects were age >18 years, normal IOP, open angles on gonioscopy, and normal optic nerves on examination.

### *CYP1B1* and *LTBP2* mutation screening

DNA was extracted using the illustra blood genomicPrep Mini Spin Kit from GE Healthcare (Buckinhamshire, UK), and stored at −20 °C in aliquots until required. The coding exons of *CYP1B1* were amplified using the primers listed in Table 1. Each 25 µl PCR reaction contained 2.5 µl of 10× reaction buffer with MgCl_2_ (Amersham Pharmacia Biotech, Piscataway, NJ); 10 pmol of each primer; 100 pmol/µl each of deoxyATP, deoxyguanosine triphosphate, deoxycytidine triphosphate and deoxythymidine triphosphate in Tris HCl buffer (Perkin-Elmer Corporation, Foster City, CA); 1 unit Taq DNA polymerase (Amersham Pharmacia Biotech); and 100 ng genomic DNA template. The mixture was denatured at 95 °C for 5 min and the PCR reaction was performed for 35 cycles, in a GeneAmp 9700 PCR system (Applied Biosystems, Foster city, CA), under the following conditions: denaturation at 95 °C for 1 min, annealing at 56 °C for 30 s and extension at 72 °C for 1 min. The final extension cycle of 72 °C was for 10 min.

**Table 1:** PCR primers for amplification of *CYP1B1*.

**Primer pair**	**Forward (5′-3′)**	**Reverse (5′-3′)**	**Length (bp)**
Exon 2	TCTCCAGAGAGTCAGCTCCG	CTACTCCGCCTTTTTCAGA	1230
Exon 3	GTCACTGAGCTAGATAGCCT	GGACAGTTGATTTATGCTCACC	844

Successfully amplified fragments were sequenced in both directions using the forward and reverse primers and the BigDye terminator v3.1 cycle sequencing kit (Applied Biosystems). Fragments were then run on the 3130xl Genetic Analyzer (Applied Biosystems) according to the manufacturer protocol. All the sequenced fragments were then analyzed using SeqScape software v2.6 (Applied Biosystems). PCG patients which were *CYP1B1* mutation-negative were screened for mutation in the *LTBP2* gene using primers and PCR conditions described previously [14] (Table 2).

**Table 2:** PCR primers for amplification of *LTBP2*.

**exon**	**Forward (5'-3')**	**Reverse (5'-3')**	**Annealing Temp.**
1a	CCCAGAGCAGGAGAAAGG	GGAACAGACTGTACACCTTGG	56
1b	GCCCCCTAGACTCAGAGAAG	AATCTTCCAATCCCGATTTT	58
2	AATGGCAGAGTCAGGATTCA	CTTCAGGACGCAGACTAGGA	55
3	CTGAGGCCAGGAGAGTGG	CCAGCCCCAACACCTACT	58
4	AAGCCTGGTGATTCCACATA	CACAAAGCAGGTGCTCAAC	60
5	GCGTCCAGTAGGTACTCAGC	AGCTAGGCTGCCAAGTGAG	60
6	GGGGCTGGTTATTATCCACT	GGCTGAGAAGTTGAGGGAAT	57
7	GGGATCATTCTGGGGTTCTA	CTGTGTGCCTGGTATTGACA	55
8	ACTCCCTTCTCCCCTTCTTT	ACAGACTGCACCAGCAGAG	60
9	GCTGAGAGGAGTCTGGTGAG	TGGCTTCCTCTGTCACTCTC	60
10	GGAGAGGAATCCCACTGAAT	ATCTCTGTTCCAGCAGGATG	60
11	ATTCCACTACGCCTCTTCCT	GCAGGGAAGGCTACTTCAG	58
12	ACGTGCTTATCCCAACCTG	TCTTGACCCCATATGGAAGA	58
13	AAGAGTCCACGCTTTCTGTG	ATGGCTGCTCCATAAACAAG	60
14	GTAAAGTGCCTGGCAGAATG	GGTGTATAGAGAGCTCCCAGAA	59
15	TTAGACTGGATGTGCTCCAAC	AGAGGGACCCTGTGTTCTTT	58
16	CCCCTAGGGTCTTATGCAAG	GAGACTGGTCTTCCCCTGAA	55
17	CCCACTGGGCTGACTTTAT	AGGCTGGAGTTCTGGTCTCT	56
18	GGGCCTGAGCTAGATCATTT	AAGGGCTCAGGAATTCTCAT	55
19	GGCAGCTCTCATTCTTTCCT	TGAATATGGCCAAAGAGGAG	60
20–21	CATGCAGAGTGCTCTGAGTTAC	GGTCCATTTATGGGGTCTTC	60
22	TTCTAGGGAGGGGGTTTTAG	AAGCTTGTGAGCGACTCTTG	59
23–24	CCCAAGAGTCGCTCACAA	ACTCCTCGCTCCCATCTTC	60
25	CGAGCCTTTTCCTACATAAGC	CAGCACGAAGATGATGATTG	58
26–27	GGAATAGATCAAGAACCCCAGA	CTTCTTTGAAGCCTCCCTTG	59
28	TCTGTCCATTGGTTCCTCCT	TGTAGCTCCTGGTTTTGCTG	60
29–30	GGCCACTTCTTAGGGTTGTG	ACAGAAAAGGTGGAGGCAAC	58
31	GTAGGAACCGGAGGCAAG	CCTGGGGACAATCTCTGAC	58
32–33	GTGGGCTGTCAGAGATTGTC	CTACTTTGTCCCCAAACAGC	59
34	ATCTCCCAGAGGGTACCAGT	CCTGGGCGTATGTACTTGTC	60
35	TCCACAAGAATTTTATGATCCTC	TTGTCTTTTGTCTGGGAACC	60
36	TGTCCTTGAGTTGCTTGGTT	TCAGGATGATGGTGGATTGT	59

### Criteria for surgical success

Criteria for surgical success were IOP ≤21 mmHg without glaucoma medications. Qualified success was IOP of ≤21 mmHg with glaucoma medication. Surgical failure was all cases that required further surgical intervention to control IOP.

### Prediction of pathogenicity

Pathogenic characteristics of detected non-synonymous sequence changes (those that change an amino acid in the resultant protein) in *CYP1B1* or *LTBP2* were assessed through evaluation of interspecies conservation and an assessment of the possible effect of the sequence change on protein function using PolyPhen [15].

### Data collection and statistical analysis

Data were collected from medical records then entered and stored in a database using Microsoft Access 2007^®^ (Microsoft Corporation, Redmond, WA). Both descriptive and inferential statistical analyses were conducted to describe different indices and determine potential associations between phenotype and genotype profiles. Statistical analysis was done using SPSS v.19 (IBM, Armonk, NY) and StatsDirect version 2.7.2 (StatsDirect, Ltd, Cheshire, UK). Odds ratios and the corresponding 95% confidence intervals were calculated to determine potential associations. Mann–Whitney U test was done to compare means of pre and post intervention indices across groups. A p value of <0.05 was used for evidence of statistical significance.

## Results

### Clinical features of the patients

Fifty four unrelated families with at least one affected member with PCG were enrolled into this study. The pattern of inheritance was consistent with an autosomal recessive mechanism of transmission in 50 families (92.6%). In 4 families (7.4%) there appeared to be a pseudo-dominance mode of inheritance and none of the families examined had an autosomal dominance inheritance. Out of the 54 families recruited, 13 (24.1%) were multiplex and in 41 families (75.9%) only one family member was affected. Consanguinity was a factor in 37 of 54 families (68.5%). In the 54 families examined, there were a total of 74 affected subjects with PCG, 38 males and 36 females. Among the PCG probands, the disease was bilateral in 20 (37%) and unilateral in 34 (63%). At the preoperative assessment, the mean (SD) age at surgery was 8.4 months (std 18.5), the preoperative IOP was 31.1 (std 9.3), with a corneal diameter of 12.8 (std 1.2), cup/disc ratio of 0.66 (0.8), and an average degree of haze of 2.0 (std 1.2) graded according to the Fantes scale [16].

### *CYP1B1* mutation analysis

Mutations in *CYP1B1* were identified in 41 (75.9%) families. No mutation in *CYP1B1* was found in 13 (24.1%) families. Among patients with mutations (n=41), 35 (85.3%) had homozygous mutations, 6 (14.7%) had compound heterozygous mutations (Table 3). We detected a total of 13 mutations: 9 missense mutations (G61E, A119S, R390H, P437L, D441G, A443G, G466S, G466D, and R469W), 2 deletions (g.4238_4247del and g.7901_7913del), and 2 nonsense mutations (R355X and R444X). Two mutations, G466S and D441G had not been previously reported (Table 3). Ten mutations were either frameshift mutations leading to premature truncation of the protein or were predicted to be pathogenic based on in silico analysis and on interspecies conservation (Table 4). Three mutations (A119S, A443G, and G466S) were predicted to be “benign.” The p.G61E accounted for 59% of mutations in probands (homozygous in 29/54 probands and heterozygous in 6/54 probands). The allele frequency for this mutation was 0.03% in controls and was heterozygous in all instances.

**Table 3:** *CYP1B1* mutation-screening in PCG patients.

** **	** **	***CYP1B1* mutations**			** **	** **
**Proband I.D.**	**Sex**	**Mutation (1)**	**Mutation (2)**	**Mutation (3)**	**Cons**	**Mode of inheritance**
109	F	g.8147 C > T	-	-	Yes	Recessive
120	M	g.3987 G > A	-	-	No	Pseudo D
237	M	g.3987 G > A	-	-	Yes	Recessive
259	M	g.3987 G > A	-	-	Yes	Recessive
262	F	g.8006 G > A	-	-	Yes	Recessive
267	F	g.3987 G > A	-	-	No	Recessive
270	M	-	-	-	No	Recessive
274	F	g.8234 G>A	-	-	Yes	Recessive
296	M	g.3987 G>A	-	-	Yes	Recessive
305	M	g.3987 G>A	-	-	Yes	Recessive
307	M	-	-	-	No	Recessive
314	M	**g.3987 G>A**	**g.4238_4247 del**	-	No	Recessive
329	M	-	-	-	Yes	Recessive
338	F	**g.3987 G>A**	**g.8233 G>A**	-	No	Recessive
494	M	g.8242 C >T	-	-	Yes	Recessive
505	F	g.3987 G>A	-	-	No	Recessive
517	F	g.3987 G>A	-	-	Yes	Recessive
556	F	g.3987 G>A	-	-	Yes	Recessive
624	M	-	-	-	No	Recessive
631	F	**g.3987 G>A**	**g.7900 C>T**	-	Yes	Recessive
638	M	-	-	-	No	Recessive
675	M	g.8167 C >T	-	-	Yes	Recessive
696	F	g.3987 G>A	-	-	Yes	Recessive
702	F	-	-	-	No	Recessive
752	M	-	-	-	No	Recessive
819	F	-	-	-	Yes	Recessive
839	F	g.3987 G>A	-	-	Yes	Recessive
844	M	-	-	-	No	Pseudo D
849	M	g.3987 G>A	-	-	Yes	Recessive
873	F	g.3987 G>A	-	-	Yes	Recessive
896	M	g.3987 G>A	-	-	Yes	Recessive
922	M	g.3987 G>A	-	-	Yes	Pseudo D
950	F	g.3987 G>A	-	-	Yes	Recessive
973	F	-	-	-	Yes	Recessive
979	M	g.3987 G>A	-	-	No	Recessive
1041	F	g.3987 G>A	-	-	Yes	Recessive
1114	M	g.3987 G>A	-	-	Yes	Recessive
1124	F	g.3987 G>A	-	-	Yes	Recessive
1125	M	-	-	-	Yes	Recessive
1130	F	-	-	-	No	Recessive
1136	F	g.3987 G>A	-	-	Yes	Recessive
1208	M	g.3987 G>A	-	-	Yes	Recessive
1246	M	g.3987 G>A	-	-	Yes	Recessive
1281	F	**g.3987 G>A**	**g.8165 C>G**	g.8159 A>G	No	Recessive
1297	F	g.3987 G>A	-	-	Yes	Recessive
1472	M	7901_7913 del	-	-	Yes	Recessive
1481	M	**g.3987 G>A**	**g.4160 G>T**	-	No	Recessive
1541	M	g.3987 G>A	-	-	Yes	Recessive
1724	M	-	-	-	Yes	Recessive
2147	F	g.3987 G>A	-	-	Yes	Recessive
2154	F	g. 8242 C>T	-	-	Yes	Recessive
2266	M	**g.3987 G>A**	**g.4160 G>T**	-	No	Recessive
2303	M	g.3987 G>A	-	-	Yes	Recessive
2333	F	g.3987 G>A	-	-	Yes	Pseudo D

Mutations in **bold** were inherited in a Heterozygous status, all other mutations were inherited in a homozygous status. Pseudo D=Pseudo-dominance; **Cons**=Consanguineous.

**Table 4:** Analysis of the non-synonymous sequence changes detected in the PCG patients.

**Nucleotide change**	**Protein change**	**Mutation type**	**Allele frequency in PCG**	**Allele frequency in controls**	**Interspecies conservation**	**PolyPhen**	**Novel**	**Reference**
g.3987G>A	G61E	Missense	0.57	0.03	High	Probably damaging	No	[8,32,40,41]
g.4160G>T	A119S	Missense	0.02	0	Low	Benign	No	[8,42]
g.4238_4247del	Del+FS	Del+FS	0.01	0	High	-	No	[6]
g.7900C>T	R355X	Nonsense	0.01	0	High	-	No	[18]
g.7901_7913del	Del+FS	Del+FS	0.02	0	High	-	No	[27]
g.8006G>A	R390H	Missense	0.02	0	High	Probably damaging	No	[18,29]
g.8147C>T	P437L	Missense	0.02	0	High	Probably damaging	No	[18,25,40]
g.8159A>G	D441G	Missense	0.02	0	Medium	Possibly damaging	Yes	This report
g.8165C>G	A443G	Missense	0.01	0	Low	Benign	No	[25,41,43]
g.8167C>T	R444X	Nonsense	0.02	0	High	-	No	[18]
g.8233G>A	G466S	Missense	0.01	0	Low	Benign	Yes	This report
g.8234G>A	G466D	Missense	0.02	0	High	Probably damaging	No	[24]
g.8242C>T	R469W	Missense	0.04	0	High	Probably damaging	No	[8,18,40]

PolyPhen pathogenicity prediction was assessed using the PolyPhen2 database. “Probably Damaging” constitutes a high confidence of affecting protein function or structure. “Possibly Damaging” reflects a likelihood of affecting protein function or structure, while “Benign” changes most likely lack phenotypic effect. “Unknown” means that PolyPhen could make no prediction due to lack of data and (-) indicate that PolyPhen is not designed to predict the effect on protein function for deletions and nonsense mutations.

### *LTBP2* mutation analysis

No mutations in *LTBP2* were found in the 13 PCG subjects who were negative for *CYP1B1* mutations.

### Phenotype-genotype correlation

We studied the possible correlation between the mutation status (present or absent) with various clinical indices at presentation and at last postoperative visit. We found that PCG cases with *CYP1B1* mutations presented with a high degree of haze than those with no mutation(s), shown in Table 5. Additionally, PCG cases with a mutation had higher last postoperative visit indices in terms of final visit haze and the need for anti-glaucoma medications. The differences in both last postoperative visit degree of haze and number of needed anti glaucoma medications were statistically significant (p=0.025, and 0.015, respectively). Comparing cases with the specific g.3987 G>A mutation to those without any mutation revealed that they were presented to hospital with a significantly higher degree of haze (p=0.027), and a higher cup/disc ratio, however, insignificantly (p=0.518). In regards to the last postoperative visit indices, cases with g.3987 G>A mutation show higher degree of haze and number of needed medication than cases without any mutation(s), where both differences were statistically significant (p=0.017 and 0.049, respectively; Table 6). Additionally, we found that among cases without mutation, the surgical success rate was 13/14 (92.9%) which was much higher than the success rate among those with mutation 42/60 (70%). This may reflect the impact of severity due to mutation where the Odds Ratio of failure among cases with mutation to those without was 5.6, however, insignificantly (p=0.110).

**Table 5:** Clinical indices at presentation and post operative for patients with *CYP1B1* mutation(s) and patients without mutation(s).

**Index**	**Cases with mutation (mean±SD)**	**Cases without mutation (mean±SD)**	**p value**
Preoperative IOP	30.59 (9.5)	33.36 (8.4)	0.322
Preoperative corneal diameter	12.74 (1.1)	13.15 (1.3)	0.260
Preoperative degree of haze	2.08 (1.1)	1.57 (1.3)	0.113
Preoperative cup/disc ratio	0.70 (0.9)	0.53 (0.3)	0.490
Preoperative number of medication	2.09 (0.6)	2.21(0.4)	0.548
Age at surgery (M)	7.61 (15)	11.21 (28.2)	0.521
**Last postoperative visit**			
IOP	16.65 (5.3)	16.43 (3.9)	0.886
Last postoperative visit corneal diameter	12.45 (1.1)	12.85 (1.1)	0.264
Last postoperative visit degree of haze	0.49 (0.9)	0.00 (0)	0.025
Last postoperative visit number of medication	0.59 (0.9)	0.00 (0)	0.015

**Table 6:** Clinical indices at presentation and post operative for patients with specific g.3987 G>A (Homo) mutation and patients without any mutation(s).

**Index**	**Cases with g.3987 G>A (mean±SD)**	**Cases without any mutation (mean±SD)**	**p value**
Preoperative IOP	30.73 (9.3)	33.36 (8.4)	0.485
Preoperative corneal diameter	12.42 (0.9)	13.15 (1.3)	0.103
Preoperative degree of haze	2.20 (1.1)	1.57 (1.3)	0.027
Preoperative cup/disc ratio	0.76 (1)	0.53 (0.3)	0.518
Preoperative number of medication	2.22 (0.6)	2.21 (0.4)	0.450
Age at surgery (M)	5.91 (14.7)	11.21 (28.2)	0.267
Last postoperative visit IOP	16.69 (5.2)	16.43 (3.9)	0.795
Last postoperative visit corneal diameter	12.35 (1.2)	12.85 (1.1)	0.077
Last postoperative visit degree of haze	0.59 (1.1)	0.00 (0)	0.017
Last Postoperative visit number of medication	0.44 (0.8)	0.00 (0)	0.049

## Discussion

We screened a large cohort of well defined Saudi PCG patients and perform detailed genotype-phenotype correlations. The mode of inheritance in the majority of the families was autosomal recessive, as may be expected in a society where consanguinity can reach up to 65% in some parts of the country [17]. Mutations in *CYP1B1* were present in over 75% of patients. As high as this rate is, it is substantially lower than that reported by Bejjani et al. [8] where the observed rate was 96% in this population. We detected 13 mutations, 11 have been previously reported in various populations [7,18] and two were novel. Three mutations (A119S, A443G and G466S) are predicted to be benign based on in-silico analysis and low interspecies conservation. Therefore, the pathogenicity of these three mutations on the development of PCG is unclear. In the literature, Certain *CYP1B1* mutations have been analyzed in-silico for their possible impact on the protein structure and function. Comparative modeling of the human CYP1B1 using the X-ray structure of CYP2c9 as a template along with molecular dynamics simulations has provided evidence for several structural differences that could impact the functional domains of this protein [19]. In vitro studies to determine the effect of *CYP1B1* mutations on the stability and function of the protein was performed by Jansson and coworkers [20]. These investigators studied the effect of two missense mutations (G61E and R469W) on the stability and enzymatic activity of CYP1B1. It was observed that the G61E mutant had lost 60% of its stability, while the R469W mutant retained about 80% of the stability compared to the wild type. The effects of the mutants on the function of protein were further determined by an enzymatic assay that further confirmed their decreased metabolic activity (50%–70%) for all the substrates when compared to the wild protein [20]. Similar studies for the variants described as “benign” by in silico analysis would improve our understanding of these variants.

We detected *CYP1B1* mutations in 75.9% of our PCG probands, whereas 24.1% had no mutation(s). This rate (75.9%) is higher than the rate observed in less homogeneous populations where consanguinity is rare or less common like Japan (20%) [21], China (17.2%) [22], Indonesia (33.3%) [23], India (44%) [24], Brazil (50%) [25],Turkey (42.8%) [26], Morocco (46.5%) [27], Australia (21.6%) [28], France (48%) [29], and Spain (34.2%) [30]. However, the rate observed here was comparable to the rates observed in Iran (70%) [31], Kuwait (70.6%) [32], and the Slovakian gypsies (100%) where consanguinity is more common [33]. It is interesting to observe that *CYP1B1* appears to play a larger role in PCG in more homogenous and inbred populations.

The G61E mutation was the most common mutation detected (63% among PCG patients). The high frequency of the G61E is likely a founder effect. This mutation was detected at a 0.03% allele frequency in 50 normal controls. Since the G61E mutation in CYP1B1 was found in 63% of the tested PCG patients and constitutes over 80% of the total mutations detected in this population, screening for this mutation first would be appropriate.

We screened PCG patients which were *CYP1B1* negative for *LTBP2* and we found no mutation(s) after screening the full gene. Ali et al. [14] first reported that the *LTBP2* mutations c.412delG, c.895C>T, c.1243–1256del, and c.331C>T caused PCG in four consanguineous families from Pakistan and in persons of Gypsy ethnicity. Narooie-Nejad et al. [34] subsequently reported two *LTBP2* loss of function mutations in Iranian families with PCG, homozygosity for the deletion c.5376delC in exon 36 and homozygosity for the deletion c.1415delC in exon 7. Although double heterozygosity (i.e., heterozygosity for a mutation at each of two separate genetic loci) for a *CYP1B1* mutation and an *LTBP2* mutation were reported by Azmanov et al. [35], the observed combination is of no clinical significance and digenic inheritance is unlikely. Apart from these reports, no further studies showing *LTBP2* mutations in PCG patients had been reported thus far. The negative results obtained here and the lack of further evidence that *LTBP2* mutations are involved in the pathogenesis of PCG raises questions about the role of this gene in PCG.

Hollander and coworkers [36,37] correlated *CYP1B1* mutations with the degree of angle dysgenesis observed histologically, as well as disease severity in terms of age at diagnosis and difficulty in controlling IOP in six congenital glaucoma patients. Their findings suggest that *CYP1B1* mutations may produce allele associated histological findings that may be correlated with disease severity. We were unable to identify any phenotypic traits that correlated with *CYP1B1* mutations or between the most common G61E mutations and other *CYP1B1* mutations. This may be due to the fact that effects are small or that this study lacked the power to determine differences.

In our study, we found that PCG cases with *CYP1B1* mutations had a last postoperative visit degree of haze and a greater cup/disc ratio than those with no mutation. In particular, the last postoperative visit degree of haze and number of medication were zero for cases without mutation(s). Moreover, PCG cases with the G61E mutation show even higher rate of the same indices. Additionally, we found that among cases without mutation, the surgical success rate was much higher than the rate among those with mutation. To the best of our knowledge, this is a novel and unique observation which might be valuable in term of predicting the severity of the disease during an early stage, choosing the best treatment regimen and surgical procedure and in providing counseling for families with affected children.

In general, genotype-phenotype correlation is highly variable. Walton and coworkers [38] have shown that the phenotype can vary significantly in the same individual (one eye being more severely affected than the other). No consistent correlation has been observed between the severity of the glaucoma phenotype and the molecular *CYP1B1* genotype among individuals with identical mutations within the same family [8], and among families with identical mutations [6,8]. No information is available on correlation between the success of surgical therapy and the type of *CYP1B1* mutation detected. However, it has been reported that patients with *CYP1B1* mutations need more surgical procedures to control intraocular pressure than individuals with congenital glaucoma without *CYP1B1* mutations [39].

In summary, this report confirms that recessive mutations in *CYP1B1* are the predominant cause of PCG in the Saudi population with p.G61E as the dominant disease-associated allele. While over 75% of PCG patients carried *CYPB1* mutations this was substantially less than previous reports. This finding suggests that this population may be powerful to detect novel genes that cause PCG. Additionally, PCG cases with a mutation had higher last postoperative visit indices in terms of postoperative haze and the need for anti-glaucoma medications. Genetic testing for patients suspected of PCG would be highly beneficial in term of predicting the disease severity. Testing for families with previous history of PCG would be beneficial and can provide a sense of relief from uncertainty and help people make informed decisions about managing their health care.
